# Fréquence, implication clinique et valeur pronostique de la lymphopénie au cours du lupus érythémateux systémique: étude cas témoin

**DOI:** 10.11604/pamj.2015.21.3.5593

**Published:** 2015-05-04

**Authors:** Fatima Zahra Ha-ou-nou, Lamiaa Essaadouni

**Affiliations:** 1 CHU Mohammed VI, service de médecine interne, Marrakech, Maroc

**Keywords:** Lupus érythémateux systémique, lymphopénie, lymphocytes, systemic lupus erythematosus, lymphopenia, lymphocytes

## Abstract

Le lupus érythémateux systémique (LES) est une maladie auto-immune dotée d'un grand polymorphisme clinique et caractérisée par la production d'une grande variété d'autoanticorps. Sa définition repose sur les critères de l'ACR dont fait partie la lymphopénie. Afin de déterminer s'il existe une corrélation entre la présence de la lymphopénie d'une part et les manifestations cliniques, immunologiques et l'activité du lupus érythémateux systémique d'autre part, nous avons réalisé une étude rétrospective, comparative portant sur 148 cas de LES colligés dans un service de médecine interne entre 2006 et 2012. Ces patients ont été subdivisés en 2 groupes: Groupe 1 avec lymphopénie (taux de lymphocytes < 1500/mm^3^) et groupe 2 sans lymphopénie (taux de lymphocytes ≥ 1500/mm^3^). Les 2 groupes ont été comparés en fonction de la présentation clinique et immunologique et l'activité de la maladie mesurée par le SLEDAI (Systemic Lupus Erythematosus Disease Activity Index). L’âge moyen des patients (134 femmes et 14 hommes) était de 35,64 ans. L'atteinte hématologique était présente dans 81,1% des cas avec une lymphopénie dans 69,2% des cas. Une association statistiquement significative était notée entre la lymphopénie et l'atteinte rénale (p = 0,025), l'atteinte cardiaque (p = 0,004), l'anémie hémolytique (p = 0,020), la présence d'anticorps anti DNA (p = 0,046), le traitement par cyclophosphamide (p = 0.035) et l'activité de la maladie (p < 0,01). En revanche il n'y avait pas de corrélation entre la présence de lymphopénie et les atteintes cutanées, articulaires et neuropsychiatriques. La lymphopénie est une manifestation fréquente du lupus systémique. Notre étude a démontré que sa présence est associée à plusieurs manifestations cliniques graves dont les atteintes cardiaque et rénale. Ceci pourrait faire d'elle un outil utile dans l’évaluation du pronostic de la maladie.

## Introduction

Le lupus érythémateux systémique (LES) est une maladie auto immune, caractérisée par une grande variété d'expression aussi bien clinique que biologique. De pathogénie encore non élucidée, sa définition repose sur les critères de l'ACR [1]. Si l'atteinte hématologique, un des principaux critères biologiques de l'ACR, a fait l'objet de nombreuses publications [2, 3], rares sont celles qui se sont intéressées de manière exhaustive à la prévalence de la lymphopénie au cours du LES, et surtout à sa signification clinique et pronostique. L'objectif de notre étude est de déterminer la fréquence de la lymphopénie au cours du LES, et d’étudier sa corrélation avec les différentes manifestations cliniques, sérologiques et activité de la maladie.

## Méthodes

**Type d’étude**: nous avons réalisé une étude rétrospective, comparative, non randomisée, portant sur les dossiers des patients hospitalisés pour LES au service de médecine interne du centre hospitalier universitaire de Marrakech, entre Janvier 2006 et Décembre 2012. Tous les patients ont au moins quatre critères de l'ACR.

**Groupes et variables**: ces patients ont été divisés en deux groupes selon l'existence (Groupe 1) ou non (Groupe 2) d'une lymphopénie. Cette dernière était définie selon les critères de l'ACR par un taux de lymphocytes inférieur à 1500 éléments/mm^3^. Le taux de lymphocytes choisi était celui réalisé au moment du diagnostic. Ont été exclus de l’étude tous les patients ayant une lymphopénie pouvant être liée à une infection sévère ou imputable à un traitement immunosuppresseur. Les patients du groupe 1 ont été classés en 3 sous-groupes: un sous-groupe « lymphopénie légère»: dans lequel ont été inclus les patients ayant une lymphopénie comprise entre 1000 et 1500 éléments /mm^3^; un sous-groupe « lymphopénie modérée »: constitué de patients avec taux de lymphocytes compris entre 500 et 1000 éléments /mm^3^; un sous-groupe « lymphopénie sévère »: comportant des malades dont le taux de lymphocytes est inférieur à 500 éléments / mm^3^. Les caractéristiques épidémiologiques, cliniques, biologiques et thérapeutiques de chaque patient ont été analysées en se fondant sur une fiche de renseignements. Nous avons adopté le système «Systemic Lupus Erythematosus Disease Activity Index» (SLEDAI) pour évaluer le score d'activité du LES au moment du diagnostic de la maladie [4].

**Analyse statistique**: la saisie et l'analyse statistique ont été réalisées sur logiciel SPSS version 13. Elles ont fait appel à deux méthodes d'analyse statistique: analyse univariée; en utilisant des pourcentages, des moyennes et des écarts- types, et analyse bivariée; au cours de laquelle, nous avons utilisé des tests statistiques notamment le test khi2 de Pearson, le test de Student et dans certaines situations le test exact de Fisher. Le seuil de signification a été fixé à 5%.

## Résultats

### Données générales

Dans notre étude 148 patients ont été inclus, dont 134 étaient de sexe féminin soit un sexe ratio H/F de 0,10. La moyenne d’âge au moment du diagnostic était de 35,64 ans avec des extrêmes variant entre 13 et 67 ans. Les principales caractéristiques cliniques et biologiques de ces patients sont résumées dans le (Tableau 1). Les manifestations articulaires ont dominé le tableau clinique avec une fréquence de 89,2%. Il s'agissait de polyarthralgies sans arthrite touchant essentiellement les petites et moyennes jointures (inter phalangiennes proximales et distales, métacarpo-phalangiennes et poignets) dans 89% des cas, 12 patients ont présenté une authentique arthrite. Cent vingt cinq patients (84,5%) avaient une atteinte cutanée, représentée par une photosensibilité chez 82 patients, un érythème malaire dans 50% des cas, et une alopécie chez 75 patients. L'atteinte rénale était observée chez 47,3% de nos malades. L’étude anatomopathologique de la ponction biopsie rénale réalisée chez 65 d'entre eux, a permis d'objectiver une néphropathie classe I de l'OMS dans 6 cas (9,2%), classe II dans 11 cas (17%), classe III dans 14 cas (21,6%), classe IV dans 26 cas (40%), classe V dans 6 cas (9,2%) et classe VI chez 2 patients (3%). Chez 5 malades la ponction biopsie rénale n'a pu être réalisée en raison du décès précoce (3 patients) ou de transfert en unité de néphrologie pour insuffisance rénale nécessitant le recours à la dialyse (2 patients). L'atteinte cardiaque présente dans 18,2% des cas, était représentée essentiellement par la péricardite lupique chez 19 malades (12,8%). La myocardite, diagnostiquée par échographie cardiaque, était observée chez 9 patients (6%). Un patient avait l'association d'une péricardite à une myocardite. Trente six malades avaient une atteinte neuropsychiatrique soit 24,3%. Les principales manifestations neurologiques étaient centrales (63,8%) à type de: convulsion chez 10 patients, troubles psychotiques chez 8 malades et de céphalées chez 5 patients. L'imagerie par résonnance magnétique réalisée chez ces patients objectivait: des lésions de démyélinisations (6 patients), une thrombophlébite cérébrale (1 patient), une vascularite cérébrale (un malade), une myélite transverse (un malade). Les manifestations neurologiques périphériques étaient rapportées par 13 malades représentées par: douze cas de neuropathies périphériques et un cas de polyradiculonévrite aigue. L'hémogramme réalisé chez tous nos patients a montré: une anémie hémolytique chez 64 cas (43,2%), une leucopénie dans 33 cas (22,3%), une neutropénie dans 18 cas (12,2%), une thrombopénie chez 29 patients (19,6%) et une lymphopénie chez 103 malades (69,6%).

**Tableau 1:** Manifestations cliniques et biologiques, caractéristiques immunologiques et traitementsdes148 patients lupiques de notre série

Manifestations	Nombre	Pourcentage
Atteinte articulaire	132	89,2%
Atteinte cutanée	125	84,5%
Photosensibilité	82	55,4%
Erythème en vespertilio	74	50%
Ulcérations buccopharyngées	34	23%
Alopécie	75	50,7%
Atteinte rénale	70	47,3%
Atteinte cardiaque	27	18,2%
Atteinte neurologique	36	24,3%
Atteinte hématologique	120	81,1%
Anémie hémolytique	64	43,2%
Leucopénie	33	22,3%
Lymphopénie	103	69,6%
Thrombopénie	29	19,6%
Anticorps anti nucléaires	122/131	93,1%
AC ani DNA	99/124	79,8%
AC anti SSA	38/120	31,6%
AC anti SSB	19/120	15,8%
AC anti RNP	10/84	11,9%
AC anti sm	38/90	42%
APL	32/110	29%
Corticothérapie	133	89,8%
Immunosuppresseur	54	36,4%
Hydroxychloroquine	94	63,5%

**Tableau 2:** Comparaison des manifestations cliniques, biologiques et prise en charge thérapeutique des patients avec (groupe 1) et sans (groupe 2) lymphopénie

Manifestations cliniques	Groupe 1 (n = 103)	Groupe 2 (n = 45)	Valeur du p
Manifestations cutanées	89 (83,4%)	36 (80%)	0,322
-photosensibilité	57 (55,3%)	25 (55,5%)	0.981
-lupus discoïde	9 (8,3%)	1 (2%)	0.284
-ulcération buccale	27 (26,2%)	7 (15,5%)	0.156
-érythème malaire	55 (53,3%)	19 (42,2%)	0.211
-alopécie	55 (53,3%)	20 (44,4%)	0.316
Atteinte articulaire	94 (91,2%)	38 (84,4%)	0.254
Atteinte cardiaque	25 (24,2%)	2 (4%)	**0.004**
-Péricardite	17 (20,4%)	2 (5,4%)	**0,044**
-Myocardite	8 (7,7%)	1 (2,7%)	**0,034**
Atteinte neurologique	29 (28,15%)	7 (15,5%)	0.100
Atteinte rénale	55 (53,4%)	15 (33,3%)	**0.025**
**Atteintes hématologiques**			
-Anémie hémolytique	51 (49,5%)	13 (28,8%)	**0.020**
-Leucopénie	32 (31%)	1 (2,2%)	**<0,01**
-Neutropénie	17 (16,5%)	1 (2,2%)	**0.014**
-Thrombopénie	22 (21,35%)	7 (15,5%)	0.413
**Immunologie**			
-AC anti nucléaires	84/90 (93,3%)	38/41 (92,6%)	1
-AC anti DNA	72/85 (84,7%)	27/39 (69,2%)	**0.046**
-APL	20/69 (28,9%)	12/41(29,2)	0.876
Corticothérapie	94 (91,2%)	39 (86,6%)	0.221
Cyclophosphamide	36 (34,9%)	8 (17,7%)	**0.035**
Hydroxychloroquine	65(63,1%)	29 (64,4%)	0,857
SLEDAI	10,71 ± 5,29	6,31 ± 3,59	**<0,01**

### Comparaison des groupes 1 et 2

Parmi les 148 patients lupiques inclus dans l’étude, 103 malades avaient une lymphopénie, soit une fréquence de 69,6%. Le taux de lymphocytes moyen dans le groupe 1 était de 844 éléments / mm^3^ avec des extrêmes variant entre 210 éléments / mm^3^ et 1500 éléments /mm^3^. La moyenne d’âge des patients était de 35,39 ans dans le groupe 1 contre 36,2 ans pour le groupe 2 avec une différence statistiquement non significative (p = 0,29). Il existait une prédominance féminine avec un sexe ratio H/F de 0,10 et 0,09 respectivement dans les groupes 1 et 2 (p = 0.875). La comparaison des deux groupes selon les manifestations cliniques objective une association statistiquement significative de la lymphopénie avec l'atteinte cardiaque (24,2% dans le premier groupe contre seulement 4% au groupe 2, p = 0.004) aussi bien pour la myocardite (7,7% dans le groupe 1 versus 2,7% dans le groupe 2) que pour la péricardite (20,4% pour le groupe 1 contre seulement 5,4% pour le deuxième groupe), et l'atteinte rénale (53,4% et 33,3% respectivement dans les groupes 1 et 2, p = 0.025). En revanche les deux groupes étaient comparables pour les autres manifestations: articulaire (p = 0,254), cutanée (p = 0,322), et neurologique (p = 0,1). En comparant les autres anomalies hématologiques et caractéristiques sérologiques des patients des deux groupes, nous avons établi une corrélation significative de la lymphopénie avec l'anémie hémolytique (p = 0,020), la leucopénie (pFigure 1).

**Figure 1:**
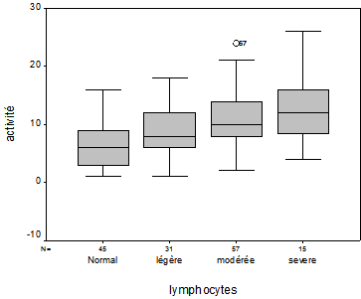
Associations entre les différentes catégories de la lymphopénie et l'activité du LES (SLEDAI) chez les 148 patients de notre série

## Discussion

Le lupus érythémateux systémique est une maladie auto-immune, touchant préférentiellement la femme jeune et évoluant par poussées. Doté d'un grand polymorphisme clinique et biologique, sa pathogénie demeure peu claire [1]. Dans la littérature, de nombreuses séries de LES ont été rapportées de part le monde, se focalisant surtout sur son épidémiologie et ses atteintes les plus sévères telles l'atteinte rénale et neurologique. Toutefois, peu d’études se sont intéressées à la lymphopénie, une manifestation non seulement fréquente mais dont l'implication peut dépasser le simple diagnostic. [5] Observée chez 69,6% des patients de notre série, la prévalence de la lymphopénie est comparable aux autres séries où elle est observée dans 47,3 à 81,74% des cas [3, 5–8]. Certaines études ont montré que cette fréquence était variable selon l’âge, le sexe et l'origine ethnique. Ainsi, la lymphopénie semble être plus fréquente chez les hommes que chez les femmes et se produit plus fréquemment chez les patients d´âge avancé et d'origine non caucasienne [9–11]. L´utilité clinique de la lymphopénie dans le LES a été limitée principalement à faciliter le diagnostic, puisqu'elle constitue un des principaux critères hématologiques de l´American College of Rheumatology (ACR). Cependant, à travers ce travail, nous avons pu démontrer que la lymphopénie était associée à certaines manifestations telles l'atteinte rénale, l'atteinte cardiaque (péricardite et myocardite), la leucopénie, l'anémie hémolytique et la présence d'anticorps anti DNA, mais également est corrélée avec l'activité de la maladie. La corrélation entre la lymphopénie et les manifestations cliniques du LES en particulier les atteintes neuropsychiatriques et rénales, a été rapportée par quelques travaux. En effet, dans une étude longitudinale portant sur 591 patients, Vila et al. ont démontré une forte corrélation entre la lymphopénie et l'atteinte rénale ce qui rejoint nos résultats [5]. Yu et al., dans leur étude rétrospective de 186 cas de lupus pédiatrique, ont trouvé des résultats en faveur d'une prédisposition à l'atteinte neuro-psychiatrique en présence d'une lymphopénie marquée [7]. Divers mécanismes pathogéniques ont été avancés pour expliquer ces corrélations, notamment une augmentation des anticorps anti protéine ribosomale P qui sont non seulement toxiques pour les neurones mais également pour les lymphocytes induisant leur apoptose [12]. De plus, les patients atteints de LES avec glomérulonéphrite proliférative présentent des titres élevés d´anticorps anti-cellules T qui seraient à l'origine d'une lymphopénie [13].

La corrélation entre la lymphopénie et l'atteinte cardiaque n'a pas été décrite auparavant. Aujourd'hui, une des causes majeures de mortalité au cours du LES est la mortalité cardio vasculaire. Une patiente lupique de 40 ans a 50 fois plus de risque de faire un infarctus de myocarde comparée à une femme du même âge bien portante. Ce risque cardio vasculaire accru a une double origine: une tendance prothrombotique liée essentiellement à la présence d'anticorps anti phospholipides, et une maladie athéromateuse précoce et évolutive [14]. Les causes de survenue de cette dernière sont multiples telles l’âge, l'hypertension artérielle, l’état inflammatoire et plus récemment la lymphopénie [15, 16]. En effet, dans leur étude longitudinale portant sur 76 cas de LES juvénile, Huang et al. ont démontré que la lymphopénie était le seul facteur de risque indépendant de progression d'athérosclérose infraclinique [17]. En ce qui concerne les anomalies immunologiques, notre travail est en cohérence avec les données de la littérature. L´association de la lymphopénie avec les anticorps anti-ADN est particulièrement intéressante car ces autoanticorps peuvent avoir une activité lymphotoxique par réactivité croisée entre le matériel nucléaire et la membrane des lymphocytes [18]. Une autre implication clinique importante de lymphopénie est la possibilité de prédisposition aux maladies infectieuses. Il est bien connu que les infections sont une cause majeure de morbidité et mortalité chez les patients atteints de LES. En présence d'une lymphopénie au moment du diagnostic, ces patients ont 5 fois plus de risque de développer une infection sévère dans les 6 six ans qui suivent [8]. Enfin, la corrélation de la lymphopénie avec l'activité de la maladie (SLEDAI) dans notre étude est confirmée par les autres travaux. En effet, Vila et al. ont montré qu'une lymphopénie modérée (500-999/mm^3^) et sévère (^3^) était associée à une activité plus élevée de la maladie [5]. En outre, Mirzayan et al. ont constaté que la lymphopénie constituait un facteur prédictif de plus forte activité de la maladie dans l'an qui suit [19]. Cette association avec l'activité de la maladie peut être expliquée par divers facteurs. Dans le lupus actif, l´apoptose des cellules T est augmentée en rapport avec une expression élevée de l'antigène Fas lié à la membrane et soluble [20]. De plus, les cellules T CD4 et CD8 qui portent la molécule CD28, un puissant signal de co-stimulation de l´activation des cellules T, sont diminués dans le sang périphérique des patients atteints de LES. Il semble que cette co-stimulation CD28 influence la susceptibilité des cellules T à la mort cellulaire et peut être impliquée dans la lymphopénie [21]. Sans oublier la séquestration importante des lymphocytes au niveau du site inflammatoire et organes lymphoïdes. Notre étude a permis d'authentifier plusieurs implications cliniques de la lymphopénie au cours du LES, cependant les biais liés à la non randomisation et à la non représentativité de l’échantillon pris, le manque de données sur l’évolution à moyen et à long terme et le caractère rétrospectif de l’étude, rendent nécessaire la réalisation d'autres études qui prennent en considérations ces différents points.

## Conclusion

La lymphopénie est une manifestation fréquente au cours du LES. Elle peut être associée à certaines atteintes viscérales telles l'atteinte rénale et cardiaque comme démontré dans le présent travail. Sa corrélation avec l'activité de la maladie pourrait faire de la numération lymphocytaire, examen peu coûteux facile et accessible, un outil utile pour l’évaluation de l'activité de la maladie.
